# Histology

**Published:** 1867-02

**Authors:** 


### DENTAL REGISTER.

VOL. XXI.] FEBRUARY, 1867. [NO. 2

### Original Communications.

### HISTOLOGY.

Read before the Brooklyn Dental Association, November 28, 1866.

HISTOLOGY may properly be said to be an account of the formation and growth of tissues. And is derived from two Greek words—histos, a web and logos, a discourse. And has been little better than a "hodge podge" of the crudities of the older observers, copied from one to the other, with accommodating credulity and reliance upon the correctness of the work ready performed to hand, out of which to construct text books. To such an extent has this been the case, and still continues to be, that the student or reader of these works must be as well acquainted with the subject as the authors, to enable him to understand even that which is attempted to be demonstrated.

Tissue—from texo—to weave. Tissue is made up of elements denominated, primary or anatomical. These are so small that the natural vision is incapable of detecting them. Therefore, we are indebted to artificial aid for whatever knowledge we possess respecting these small bodies.

This aid consists in the various modifications of microscopes, simple and compound, with a great variety of accessory appliances. Every department of which must be well understood and properly applied to enable us to arrive at the demonstration of the truth.

A complete understanding of Histology then, involves a knowledge of the whole range of material (planetary) science. For our survey is not complete until we have detected, understood, and explained the rise, development to full culmination in size and proportion, no less than the decadence and death of each variety of tissue in all the divisions of material (planetary) existences denominated—primalia, mineral, vegetable, animal and human.

Every tissue is made up of cells in various stages of obliteration. And every cell is but an attempt at consolidation of a portion of amorphous or chaotic matter in plastic condition. Perfect or regular consolidation is no less than crystalization. And every degree of togetherness, called consolidation, from fluid to solid, is a step in the direction of perfectly regular crystalization.

There are two causes of fluidity at exact antipodes of molecular movement to each other, viz.: *intensity* of molecular motion—fusion by heat as it has been called; and *extensity* of molecular motion—separation into gases and ethers. Whatever opposes these states of molecular movement tends to solidify the body.

The radical tripod of currental lines, upon which organology stands as a base, is an equation of chemism, electrism and magnetism.

In the primalia these radicals are inclosed in a bleb of oxydised hydrate of carbon, the periphery of which becoming dessicated, as is said, but really hyperoxidised, the exterior of the sphere (which the bleb always is when free), becomes endowed with a plus quantity of oxygen to the interior, which divides the sphere into hemispheres or concentric spheres of plus and minus or seminal and germenial qualities, by which proliferation takes place and we thus have cell development in its simplest manifestation.

The alternations of the generations of cells might have been synthetically conceived, but it could not have been analytically proven, until the microscope had been discovered and properly constructed.

Heterodox, as is all aphoristic pronouncement of truth to the partially developed and pseudo-scientific mind who tarry in the specials without being able to perceive the generals of the aspect of things, because they are unable to withdraw the mental attention from the confusion of the unimportant detail in closest proximity; nevertheless the exercise by which they differentiate any objects in the field of view from any other, is in essence the same kind of mental action, only less in degree, that would emancipate them from this narrow confinement and prove their ability to soar and see for themselves the truths they had just before denounced as "heterodox"—"*untrue!*" If to be "*sensible*," man must be superior to the domination of his mere "*senses*:" Let us attempt the equation of his bodily and mental affections.

Feeling being the base of all the senses, as sight is their sum and apex, we may say that the scientific equation of the senses may be thus stated, viz.: The five divisions in nature, as a whole, may be said to have their sentient correspondences thus: 1. Primalia to Feeling. 2. Mineral to Taste. 3. Vegetable to Smell. 4. Animal to Hearing. 5. Human to Sight.

The pyramid is built with a broad base of many stones. Each layer being reduced in regular succession of unifying refinement until we arrive at the top where one admirable, single, perfectly-wrought stone completes the form and limits the outline of its beautiful body. The *plan* is adhered to, and the *body* is perfect. So in Nature, the Primalia are the base and man the apex of the organic structures on this planet. The Orders, Classes, Families, Genera, Species and Varieties being more abundant, occult and practically innumerable in the base, but finally culminating in the single Genus and Species—Homo—in unitary type in man as the sum of all the preceding, and the apex or head of the great plan of Nature, as a whole. It is plain then that indifferentiation or sameness, and differentiation or unlikeness are the dual primates, without an understanding of which we shall not be able to classify the works of our minds or bodies so as to be able to propound them to our fellows in understandable, demonstrable shape. This brings us to the dogmatic aphorism that there is no *body* without its due proportion of *mind* as certainly as that there is no segregate perception of things without body, in which the mind, or seat of perception, has its focalization, or proper residence.

As material science deals only with the statical aspects of things, it becomes necessary for us to advance into the region of dynamics proper, the field in which matter is produced out of substance and transmuted into all the possibles of sameness and unlikeness, or togetherness and apartness; the various stages and degrees of which constitute every possible variety of tissue throughout the entire range of seen and unseen organic being.

Transparency and opacity being but the degrees of satisfaction or dissatisfaction of type, with residence or tenant with tenement; instance, compatible, chemical solutions from seraph to silex, and all possibles between these astounding extremes!

That the extremes are astounding to us, all will agree, but, that there is any real relation between them, I have not the confidence to hope that many will admit at first view. No one rejects truth, knowing it to be such; but we are apt to call that truth only which is in the garb of some already known and established presentment thereof.

An attempt to prove the reality of relation between a burning seraph and a brilliant crystal of silex, to the mind that demands that the testimony shall all be brought down to his own particular position, while he refuses to be carried up to higher and clearer fields of survey, the traverse of which would empower him to perceive relations before out of the range of his perceptive ability; would be much like demonstrating the higher relations of number and form, so clearly provable in mathematics and geometry, by the necessary preparatory training and development of apprehensive power, to him who had grown to manhood without the advantages of a primary school, a difficult task, and a lifetime labor of love, —first, to remove all the obstacles, and then impress each step of the process, deep enough to enable him to hold the singulars near enough together, and in the proper order of relation to favor their cancellations into the general which swallows them each and all in the presentment of their rounded proportion of clear and satisfactory demonstration.

If, then, all truth, to be communicated or taught to others, must first be put forth in positiveness of statement, sometimes invidiously called "dogmatic propositions," we must expect to have it cautiously received by the truly philosophic mind, bluntly scouted and rejected by the self-sufficient dogmatic mind, and listlessly stared at by the superficial and frivolous cast of mental endowment; and so amid all these discouragements have a weary way to general demonstration and recognition. But as there must have been a time when the present splendid array of clearly proven and firmly established truth was outside of all human apprehension of its power and presence; it is evident that there must have been a will and a way by which this blank became the well-stocked book of recorded experiences upon which men of science make such multifarious interchange of that which each, in his individual researches, may have come in possession of, in fact and philosopy, which together constitute that which we call scientific attainment.

The fact is now a days we are so universally born into the world already so richly endowed with the labors of others, that we very complacently come into the splendid accumulations of the pecuniary and mental wealth attained by our predecessors, and find it much easier to accept and use it than to inquire how they became so marvellously endowed.

But if we do not learn this first lesson of their success, it is certain that the time will come when we shall be bankrupt in both money and that knowledge which teaches us how to retrieve our lost fortunes.

To get at general propositions such as the tremendous equations of greatest differences, we must transport ourselves mentally back to the time when the morning stars sang their first symphony, immediately after darkness and nonentity had been dissipated by the power of that voice which said "let it be," and lo it stood forth, first in elemental chaos out of which each form then took serial order from highest to lowest and lowest to highest. Another dogmatic formula of *power* and *presence*, or dynamic and material correspondences, will enable us to apprehend more exactly the degrees of mental labor or the process by which we work up into our *intellects* the *feelings* that spontaneously arise in and pervade our sentient natures. By going one step below the primalia (or feeling) and one above man (or sight), we are enabled to produce the section of a complete human sentient centre or the exact formula of mental operation, viz.; 1. Chaos (corresponds by antithesis) to knowledge (demonstration). And here we repeat the five senses and their co-relatives, with the single difference of beginning with chaos as unity, thus: 2. Primalia to Feeling. 3. Mineral to Taste. 4. Vegetable to Smell. 5. Animal to Hearing. 6. Man to Sight. 7. Supernal (Sentiency) to Inspiration. By adding chaotic, and supernal states to the former diagram or section we are enabled to perceive in this corrected one how we are connected above and below, and thus get out of our mere senses into states of definite and indefinite apprehension whereby to measure curselves and correctly take our true position in the universe, physically, mentally and morally.

It was stated that "chaos" (matter in its strictest sense), "by *antithesis*," "corresponded to knowledge." From an exterior stand point this is true, but if we view it as a mental process occurring in the individual and capable of repetition upon every impulse inspirationally produced, we must reverse the order of numerical annotations to enable us to correctly observe the process of an inspiration becoming a knowledge or overpowering perception of the truth! Let us say that the individual is formed and ready to perform the first act of mentality. A sense of need, deficiency, instructually comes upon him, which we denominate aspiration, this induces 1. Inspiration which strikes the periphery of the mind or sentiency, causing undulations denominated 2. Feeling, this when culminated produces 3. Idea, which in like manner of gestational activity becomes 4. Thought, and by continuance this merges into 5. Opinion, and here the impetus being regularly continued in due process of movement this merges into 6. Belief, and this by simple intensification of the same centripetal action fuses into the complete, clear and incontrovertible demonstration of 7. Knowledge. Thus knowledge is the indisputable property and most interior of the individual mind. Deficiency and fullness are then most interior states and upon these will our activity or lethargy depend.

Let us recapitulate for the sake of conciseness and clearness of definition. Attainment of knowledge is a centripetal direction of mental labor. Forgetting is a centrifugal course of mental action. Coming to us the first work is (1) Inspiration, (2) Feeling, (3) Idea, (4) Thought, (5) Opinion, (6) Belief, and (7) Knowledge. Going from us, (1) Knowledge dwindles into (2) Belief, this into (3) Opinion, and this into (4) Thought, which very soon dies out into (5) Idea, which dissipates into the vagueness of mere (6) Feeling, when it ceases to be within the mental grasp the instant it passes into the sphere of (7) Inspiration. And thus is lost the accumulations of a lifetime by inverting the currental movements in the channels through which the acquisitions were made. Probably the greatest difficulty in the way of exactness in all matters of science is to be found in the assumption by each individual observer of his particular position in the universe of mind and matter being the grand centre from which all observations must be taken to be tolerated as even possibly correct. Scientific matters have come to such a pass, that he who would know must either isolate himself, be a hypocrite or possess the innocency of the dove coupled with serpentine sapiency to the degree of the equation of that difficult problem of deficiency and fullness of mental wealth. Do I hear some one say, "If all this and more be involved in the attainment and correct propounding of histology in even a small department of its bewitching domain of knowledge, I fear there are few who have the courage to undertake it." To which, permit me to reply, then there are few who apprehend the direction in which the happiness of themselves and the whole race alone can be found, unless that happiness be attainable in a reversion to savagism, for until we learn how to obey law intelligently we must fall back upon mere animal instinct which would soon so reduce the population as to make even traditional science an impossibility. Waiving then for the present the formation of matter out of substance, let us contemplate a system of organs fully ripened in its tissual constituencies so as to be in possession of a plus quantity of the material riches, an exact equation of which constitutes the pabulum upon which these tissues feed. This system being fed above individual need, begins to lay by in store tissue material or food for cells proper in receptacles provided for the purpose. This hypothetical radical of chaos has two distinct forms of bodily presence—one the type of fluids and motile force, the other the type of solids or statical receptivity—*i. e.*, active and inactive—masculine and feminine, seminal and germinal, &c., &c., or any other name by which the inter-dependent necessities of positivety and negativety may be called.

All bodies floating free in a medium rarer than themselves are necessarily endowed with an equator, poles and zones, in which the resistence of the medium to the rotating and orbital force produces currents in exact agreement with the equation of these forces.

In case the body be hard and resistent the currents will be on the surface, in the medium; and in case of being fluid they will be on the surface and in the body of the fluid, to a depth varying with the circumstances of the number of the currents in the body as a whole or a planet, and with other modifying conditions. That is a dense medium and rapid orbital (which is the concomitant of rapid revolving), motion will produce rapid and numerous currents upon the seas, so to speak, the motion raising them, the seas, at their margins like the edges of whirlpools and depressing them in their centers.

The diversity of the molecular motions (heat) at the poles and the equator divides the body into hemispheres in which the effort at equalization of molecular motion (heat) becomes the prime cause of the currents from the poles to the equator and from this to the poles which constitute the currents in circular or eliptical form in accordance with the resistances they meet with defining them as circles or elipses. In case of rarity of medium and slowness of movement of the body, these currents will pervade larger surfaces of the seas and move with less rapidity therein and thus necessarily be fewer on the planet and of less proportional depth of center and elevation of margin or edge.

As a sequence of the basal aphorism, "all things differ but in degree," concrete and discrete, it follows that every independant body must be a planet or a microcosm in which reside all the dynamical, physical and statical possibilities, and hence be a sphere, until it ceases to be independant by becoming associated with other planets so as to interfere with its freedom; when it divides the dominion of space and accommodates itself to the change of circumstance, with the same spontaneity that it holds fulness of sphericity when without a partner or partners in the territorial space which it occupies. And it then assumes any and every form, and degree of density, necessary to accommodate itself to the divided dominion. The blood disks and lymph corpuscles are the only examples of really free bodies in the human organism, hence are the only proper spheres in the economy of "formed tissues."

The rapidity of the movements in currental lines producing primal bodies and mental processes, renders them difficult of observation; and hence they have been ignored by all but a very few who have had to be in possession of earnestness (sufficient to overcome their own deficiencies coupled with the sharp jeers of their fellows arising from the same cause, calling for "facts, more facts," before they had comprehended the significance of those already familiar to all), if they would make any progress of substantial character.

This necessity for regularity and directness of movement in both instances may be proven by a reference to the fact of quiescence being requisite to the formation of crystals and cells of slow growth; such as silex, diamond and many salts, slight agitation of the mother waters of which so effectually prevent their formation: and the failure of the mind to accomplish its work when under the influence of adverse or confusing currents.

Just so soon as the mind fails in its central attraction, a chaos of mental ebullition holds the dominion until spent or overcome by a centripetal direction being restored to the currents of motion.

I deemed it important, thus cursorily, to examine the mental tools and enabling circumstances by which these are to be brought into requisition in the investigations before us, that the steps we take may be made more certain to our apprehension. It is patent to every observer that that which we call knowledge is but fractional, not rounded out to such completeness as to commend itself alike to the uncultured and the cultured mind. This arises partly from the nature of the subject and partly from want of uniformity in manner of investigation and nomenclature. But chiefly from the false teachings that have prevailed, causing one of the investigators in this field to exclaim in the bitterness of his soul,

"Alas! the scientific *mind* is steeped in the *senses* and is the drudge of their limited sphere!" to which I would add, and so long as it remains under this dominion it will be incapable of any thing better than the accumulation of incoherent, meaningless facts without a cause or use. But if we can intelligently accept the trite aphorism, "all things differ but in degree, of concrete and discrete proportions," we will have done more to get at the clear understanding of our subject, than if we had aggregated every fact in the universe without being able to deduce the law or *plan* of nature, which they all tend to prove, the moment they are properly corelated.

And now, as the whole body derives its *force* from respiration, so also do the least as well as the largest constituents thereof depend upon the diastolic and systolic synchronism of each (breathing) to perform its proper function.

And as the entire system is at one time but a bleb—a proliferation of cell—a mere dermatozoon, the further possible evolution of which depends upon an influx of a *something* to awaken its dormant *power* into an active directing *force;* it does seem plain that all function is readily reducible to the formula of breathing (*i. e.*) inspiration (taking in) digestion (placing) and expiration (throwing out).

Then if cells are begotten, gestated, born, live out their alloted term of occupancy and dominion of time and space and then die, it is evident that many generations in alternations must live and die in the production of the tissues we are so desirous to detect in their metamorphoses into the organs out of which our personal bodies are so intricately constructed.

The eye can never settle this occult and important quest, in any but those examples of cells, tissues and organs that are in their normal, living state, quite transparent, so that the metamorphoses may be seen and recorded.

That this will not soon be accomplished in regular serial order in human organisms I dare not now assert; for it has occurred to me so often in witnessing the reproduction of lost tissues, to see vessels spring up in the transparent plasm, which occupied the former position of the primary structures, until their abundance obscured the field, that it is more than probable that the possibility of the portraiture of these successive stages is nigh upon us in some of the enthusiastic hands who now are becoming interested in the matter.

From all that I have seen, read and heard, I have no doubt that reproduction follows the rôle of production of tissues and organs in all forms of being where it takes place at all.

As this has been proven over and over again in transparent bodies, and as nature is averse to doing her work in diverse ways to bring about the same result, we are safe in setting this down as one of her settled and certain laws by which we may be guided in all our efforts to interpret the unseen by that which doth so clearly appear. The harmonious difference of chemism, electrism and magnetism, constitutes not only the origin of individual existences, but the harmonious play of every function in health. And just so soon as either has been withdrawn or supplied in excess to any degree, a pure physiology can no longer be said to hold the dominion of the cells, tissues, organs, or systems in which this state of things exists.

If then the degrees and modes of obliterations of cells constitute the differences of the tissues we meet with in original production, generation, regeneration and degenerations of all the tissues in health and disease; have we not an herculean task to accomplish if we attempt annotations and nominations for all the appreciable forms already familiar to us in what may not inaptly be called, but the dawn of histological research? That the tissues are but obliterations of cells and vessels we are now happily able to prove.

Thanks to the revealments instigated by the investigations of a singular disease, viz.: "Trichiniasis." These little parasites by the law of their nature find their way into the capillaries which supply muscular fibre and there become encysted by acting as emboli. And when not too numerous do not interfere with the function of the part longer than it requires to form new vessels and new fibres for the use of the body.

